# Psychophysiological Stress Status of Soldiers Prior to an Operative Deployment

**DOI:** 10.3390/ijerph192013637

**Published:** 2022-10-20

**Authors:** Agustín Curiel-Regueros, Jesús Fernández-Lucas, Vicente Javier Clemente-Suárez

**Affiliations:** 1 Faculty of Sport Science, Universidad Europea de Madrid, Tajo, s/n, 28670 Villaviciosa de Odón, Spain; 2 Applied Biotechnology Group, Universidad Europea de Madrid, Urbanización El Bosque, Calle Tajo, s/n, 28670 Villaviciosa de Odón, Spain

**Keywords:** heart rate variability, anticipatory stress, military deployment

## Abstract

An anticipatory stress response develops before an internal or external stimulus, which initiates a homeostasis process through a chain of responses that enable human organisms to face different threats, thus allowing them to adapt to a continuous and eliciting environment. In the current research, we analyzed the psychophysiological anticipatory anxiety response of professional soldiers prior to a real mission in an actual theater of operation. Autonomic modulation through the heart rate variability values, muscular strength manifestation, and psychological stress of 53 military personnel of Army Airmobile Forces (age: M = 35.4 years, SD = 5.88 years; height: M = 1.75 m, SD = 6.87 cm; body mass: M = 77.33 kg, SD = 11.95 kg; military duty = 14.44 years, SD = 6.43; military operation experience = 4 months, SD = 4.25 months) and a control group of 33 civil participants were analyzed. The military personnel presented significant differences in some HRV values related to the activation of sympathetic systems. We found that the military personnel presented an anticipatory anxiety response only at an autonomic level, showing an increased sympathetic modulation, but not at a psychological level, since their anxiety levels were not significantly different than those of the control civilians. In addition, this anticipatory anxiety response did not affect muscular strength manifestation, as it presented no significant differences between the military personnel and the control group.

## 1. Introduction

The stress response is one of the mechanisms that allow human beings to adapt to a continuous and eliciting environment, allowing the survival of the species [1]. New armed conflicts or recent events suffered by humanity have been decisive in the effects of stress [2,3,4,5]. An anticipatory stress response develops before an internal or external stimulus, which initiates a homeostasis process through a chain of responses that enable the organism to face different threats [6]. This mechanism for survival can manifest in the short term as an acute adaptive response, where the capacity of the organism to anticipate risks is modulated by the primitive fight–flight mechanisms [6,7], but when the stress response is activated in a chronic way, it can become pathologic [7,8,9]. 

The preparation of the organism for any threat activates anticipatory anxiety responses that lead to behavioral, cognitive, and psychological modifications [8,9,10], such as changes in motor performance [11] and harmful effects on cognitive functions such as attention, decision making or memory [9,12], or alterations in the autonomic nervous system (ANS). In this context, it was found that the sympathetic nervous system (SNS) increases its modulation while activating the preparatory response of the organism [13].

Recently, autonomic modulation has been analyzed through the study of heart rate variability (HRV). HRV is a marker that shows the activity of sympathetic and vagal components of the ANS on the sinus node of the heart [14]. Acute stress exposition has been evaluated in relation to HRV in those professionals working in environments with high-stress exposure such as police [15], medical personnel [16], firefighters [17], and military personnel [18,19]. In these different types of professionals, previous authors found that HRV response is modulated by their environment, as well as their experience, previous specific training, the task performed, and for military personnel, the type of military unit [20]. HRV was also used to measure athletes’ values, serving as a predictive method to establish their training and rest protocols [21,22], and in mental diseases to improve intervention protocols [23]. 

Specifically, in the military field, it was observed that parachute jumping produces a large sympathetic modulation among paratrooper units [24,25], as well as in warfighters in asymmetrical combat environments [26], underground operations [27], special operation courses [28], survival maneuvers [29], or fighter jet pilots in combat flight maneuvers [30,31] and helicopters crews [32,33,34]. Chronic exposure to stressful events in military units was related to the prevalence of anxiety disorders and an increase in diagnoses over time in this millennium, with a prevalence of 0.8 per 100 service members for first service occurrence, followed by post-traumatic stress disorder (PTSD) at a rate of 0.5 out of every 100 [35]. In this context, the predominance of PTSD diagnosis has increased in recent years due to recent armed conflicts such as those occurring in Afghanistan or Iraq [36,37,38,39]. PTSD is related to trauma and stress and occurs as a result of experiencing a traumatic event, which leads to recurring memories and dreams, amnesia, social isolation sleep problems, among other symptoms [40,41].

In the present study, we aimed to answer the question of how real military deployment affects the anticipatory anxiety response of military personnel. 

## 2. Materials and Methods

### 2.1. Participants

The data of a total of 53 military personnel of the Spanish Army Forces were analyzed during the week before operative deployment in an actual theater of operation in 2019 (age: M = 35.4 years, SD = 5.88 years; height: M = 175 m, SD = 6.87 cm; body mass: M = 77.33 kg, SD = 11.95 kg; military duty = 14.44 years, SD = 6.43; military operation experience = 4 months, SD = 4.25 months) and a control group of 33 civil participants in military draft age, physically active, and without physical or psychic pathologies (age: M = 28.64 years, SD = 9.10 years; height: M = 1.74 m, SD = 8.98 cm; body mass: M = 73.42 kg, SD = 12.12 kg). All the research procedure was explained to the participants prior to the study, and all the participants filled out an informed consent form following the Helsinki Declaration. In addition, all the procedures were approved by the local ethics committee CIPI/18/093 on 6 March 2019. 

### 2.2. Procedures

To reach the study aim, different evaluations were made in order to analyze the anticipatory anxiety response. We measured the quantitative variables of autonomic modulation and muscular strength manifestation and the qualitative variables of the participants’ anxiety levels. The participants were sought within the unit that was going to be deployed who did not have an illness, did not take medication, and had no hand injuries. All the variables were evaluated one week before deployment.

### 2.3. Autonomic Modulation

Autonomic modulation was studied through the analysis of the HRV of the participants. HRV was evaluated using a validated Polar V800 heart rate monitor (Polar, Kempele). The participants remained seated for 5 min in a quiet room with a Polar electrode belt placed in the middle of the chest following Polar’s guidelines [42] and previous research in this population [43]. 

The following HRV variables were obtained:

(a) The time domain features: mean R–R; the standard deviation of successive differences (SDNN); mean HR; the root mean square of successive differences (RMSSD); and the number of adjacent intervals varying by more than 50 ms (PNN50);
(b) The geometric measure: interval histogram (TINN);
(c) The frequency domain features: the low-frequency band in normalized units (LF n.u); the high-frequency band in normalized units (HF n.u); and the ratio between the low- and high-frequency band LF/HF ratio.

### 2.4. Muscular Strength Manifestation

Immediately after the HRV evaluation, muscular strength manifestation was evaluated. The isometric hand strength (IHS) in the dominant hand of the subjects was tested using a dynamometer. The participants remained in a standing position with their arms extended and performed two contractions with their dominant arm, taking the highest data as the result.

### 2.5. Anxiety

The anxiety response of the participants was evaluated using the Spanish-validated version of CSAI-2R with a CFI (comparative fit index) and NNFI (non-normalized fit index) value of 0.97 and an RMSEA (root mean square error of approximation) index of 0.045 [44] used in previous military research [19], which consists of 17 items that analyze cognitive anxiety (CA), somatic anxiety (SA), and self-confidence (SC).

Along the same line, the state anxiety was evaluated using the 20 state-anxiety-specific items of the State-Trait Anxiety Questionnaire (STAI) [45] validated as a simple, brief, and useful self-report for the assessment of anxious symptomatology [46] and used in previous military research [47]. 

### 2.6. Statistical Analysis

The IBM SPSS statistical package (version 21.0; SPSS, Inc., Chicago, IL, USA) was used to analyze the variables. Descriptive statistics (mean and standard deviation (SD)) were calculated for each variable. Then, the Welch test was used to analyze the differences between the groups in the variables studied. The level of significance for all the comparisons was established at *p* ≤ 0.05.

## 3. Results

The results are reported with the mean and standard deviation. Table 1 shows the psychophysiological values obtained in the military personnel and control group. The army personnel presented significantly higher values in LF (n.u.) and LF/HF and significantly lower values in HF (n.u.), SA, and CA than the control group. No significant differences were found between the groups in terms of other parameters.

## 4. Discussion

The objective of the present research was to analyze the psychophysiological anticipatory anxiety responses of professional soldiers prior to a real mission in an actual theater of operation. The initial hypothesis was partially fulfilled since the soldiers presented anticipatory anxiety responses prior to deployment only in the autonomic nervous system response but with no psychological manifestations.

The analysis of HRV allow us to know the autonomic response of participants [48,49,50,51,52]. Analyzing autonomic modulation, the activation of the sympathetic nervous system was observed in the military group. The autonomic modulation behavior was also measured in previous studies involving civil and different military populations [53,54,55,56,57], such as those in symmetrical combat, asymmetrical combat [27], and tactical parachute jumps [24,25], as well as helicopter pilots before and after different rescue flight maneuvers [34,58]. In this line, the sympathetic modulation found in these studies was higher than that evaluated in the present one. This difference could be related to the fact that in the other studies, the participants faced the threat stimuli immediately; in this situation, the activation of the flight–flight response was higher, and the sympathetic modulation was also higher [19,59,60]. In the present research, by contrast, the threat stimuli were not immediately present; therefore, the activation level was lower. The increased sympathetic response due to eliciting stimuli was also observed in other stress contexts such as in sports, where ultra-endurance runners showed a sympathetic modulation increase during these extreme races [61]. It was also measured in educational contexts, where a high anticipatory anxiety response was also revealed at the beginning of laboratory practices in biomedical science students [62]. Another collective for whom sympathetic modulation was evaluated and a high level of activation was found involved the healthcare professionals, with studies showing a high level of activation of the sympathetic system in student nurses in their first hospital clinic practice [63] and, in the same context, during the hospital clinical simulation practice [64].

In this line, it was found that the military group presented lower RMSSD values than the control group but not significantly. Lower RMSSD values correspond to the activation of the autonomic sympathetic nervous system [48,49,50]. This tendency in the behavior of this parameter could be related to the sympathetic activation induced by the anticipatory anxiety response since the organism presupposes a future threat and prepares the body’s systems to face that threat [8]. Additionally, significant differences were found in the HRV frequency domain parameters. The military group showed significantly higher LF values and significantly lower HF values than the control civilians. In this line, the LF/HF indices of the soldiers were significantly higher than those of the civilian group, which corresponded to the activation of the sympathetic nervous system, corroborating the anticipatory anxiety response of the military personnel [51]. This condition showed that the anticipation of the eliciting scenario, i.e., the theater of operation, leads to a decrease in parasympathetic activity, which is related to a decrease in the HF values and an increase in the LF/HF and LF values [52]. This modification in the HRV frequency domain values was also shown in different other operative scenarios such as special operation teams before a combat simulation [53] or in military pilots before an operative maneuver [30]. It was also shown in nurses during hospital stays [54]. This activation of the anticipatory anxiety response shown by military personnel provides the organism with a state of alert that prepares it for any possible threats and contexts even if the stressful event is not immediate [55]. This fact was observed in pre-competitive sportspeople such as runners [56] or athletes from the BMX discipline [57]. 

Independently of the activation of the sympathetic nervous system found in the military personnel, it was also found that both groups (military and control) presented percentiles in the psychological questionaries considered normal in their age groups. In this line, the control group presented significantly higher levels of somatic and cognitive anxiety than the military group, although the control group’s values were characterized as normal levels of anxiety. The lower manifestation of psychological stress presented by the army personnel should be due to the preparation, training, and academic preparation of military personnel to face this kind of event [65]. This fact was observed in military pilots before a real and simulated flight maneuver [30] and could be modulated by the experience of soldiers [53].

In the same case, there were no significant differences between the groups in terms of muscular strength manifestation. Previous studies found that muscular activation occurs due to an anticipatory anxiety response [66] and an increase in strength manifestation was noted during the flight–flight system in other types of army operations [29]. The muscular activation presented in this research was lower than that shown in infantry personnel before operative training [67] and lower than that of parachute jumpers before high-altitude low-opening and high-altitude high-opening parachute jumps [25,59]. Along the same line, the values presented by the military group in this research were lower than those reported for the military personnel during melee combat [60]. This fact could be because the threat stimuli presented in this research were not high enough to result in muscular activation [68,69,70].

### Limitations of the Study and Future Research Lines

The main limitation of this study was the lack of a large group of military personnel who were evaluated, and no distinction was made about gender or mission experience. Another limitation is that the control group was not equal in number to the experimental group, and no distinction was made about the type of profession.

Creating a database regarding the type of training, protocol deployments, and the differences in physiological responses between genders and military forces could be useful in future studies to track psychophysiological responses before and after a mission. In this context, it could be beneficial to know the psychophysiological state after a military deployment.

## 5. Practical Applications

The knowledge of the anticipatory response of anxiety presented in this research adds to the previous studies that associate high levels of anxiety with different psychic pathologies and could be useful for the creation of intervention programs to improve the autonomic modulation of military personnel.

## 6. Conclusions

We found that the military personnel presented an anticipatory anxiety response only at an autonomic level, showing an increased sympathetic modulation, but not at a psychological level, since their anxiety levels were not significantly different than those of the control civilians. In addition, this anticipatory anxiety response did not affect muscular strength manifestation, as it presented no significant differences between the military personnel and the control group.

## Figures and Tables

**Table 1:** Psychophysiological parameter differences between military personnel and control group.

	Military Personnel		Control Group		*p* Welch Test
Psychophysiological Parameters	M	SD	M	SD	
IHS (N)	44.85	10.90	40.81	8.06	0.088
HRV parameters					
Time domain features					
Mean RR (ms)	910.43	129.21	874.65	154.68	0.283
SDNN (ms)	96.75	43.33	79.53	123.30	0.458
Mean HR (bpm)	68.29	9.57	70.85	13.59	0.096
RMSSD (ms)	47.40	17.15	60.55	40.20	0.360
pNN50 (%)	20.72	12.36	27.78	21.00	0.096
Geometrical feature					
TINN (ms)	325.00	141.36	316.35	140.22	0.787
Frequency domain features					
LF (n.u.)	77.06	13.38	62.63	15.73	0.000
HF (n.u.)	22.91	13.37	37.31	15.68	0.000
LF/HF ratio	6.05	6.69	2.27	1.57	0.000
Anxiety parameters					
CA (-)	8.98	2.80	10.91	3.75	0.036
SA (-)	10.30	2.45	11.83	2.90	0.037
SC (-)	17.93	2.33	16.70	2.80	0.076
STAI A/S	10.53	7.70	13.60	7.13	0.106

M: mean; SD: standard deviation; IHS: isometric hand strength; SDNN: standard deviation of normal-to-normal R–R intervals; RMSSD: square root of the mean of the sum of the squared differences between adjacent normal R–R intervals; pNN50: the percentage of differences between R–R intervals higher than 50 ms; HRVI: HRV triangular index; TINN: interval histogram; LF: low frequency; HF: high frequency; n.u. CA: cognitive anxiety; SA: somatic anxiety; SC: self-confidence; STAI A/S: State–Trait Anxiety.

## Data Availability

All the data are presented in the study.
